# Large B-cell lymphomas

**DOI:** 10.1097/HS9.0000000000000272

**Published:** 2019-06-30

**Authors:** Umberto Vitolo

**Affiliations:** University Hospital Città della Salute e della Scienza, Turin, Italy

**Learning goals**

After attending this session, you will be able to:

- Recognize the molecular heterogeneity of large B-cell lymphomas and understand current molecular classifier.
- Learn how new treatment strategies can be developed based on identified target alterations.
- Learn which prognostic and predictive biomarkers are biologically meaningful, robust, reproducible, widely available and affordable to serve as companion diagnostics.
- Recognize the optimal treatment for your DLBCL patients, learn the most recent data on the integration of novel drugs to standard chemmoimmunotherapy and discuss how new combo strategies, based on molecular and biological DLBCL subsets, may improve the outcome of DLBCL.

## Introduction

Diffuse Large B-cell lymphoma (DLBCL) is the most common lymphoid malignancy. Despite its high curable rate, 60%, with Rituximab-CHOP, 40% of the patients did not respond or relapse. To improve their outcome, we need to learn more about the heterogeneity and complexity of DLBCL. This educational session is focused on molecular and pathological subtyping of aggressive lymphomas and how treatment can be driven accordingly.

Dr Chapuy will review genetic analysis of DLBCL. Gene recurrent mutations, somatic copy number alterations and low frequency alterations has been studied leading to the identification of five distinct molecular DLBCL subsets and other similar models. These genetic features provide new insights in the DLBCL pathogenesis, predicting the outcome and may lead the pathway to novel combination treatment approaches.

Cell of origin (COO) has been extensively studied in DLBCL with activated B-cell-like subgroup carries the worst prognosis. The underlying genetic differences have been shown to differ between COO subgroups although with considerable overlap. Double hit lymphomas with MYC, BCL2 and/or BCL6 have a dismal prognosis and their recognition is mandatory because of different treatment strategy. The expression of predictive biomarkers as MYC, BCL2, CD30 and other is also important with several uncertainties. Dr de Jong will review all these histopatological findings.

Based on these premises, many expectations have been placed on clinical trials using novel compounds as Ibrutinib, Lenalidomide, Venetoclax and others with R-CHOP, based on COO classification or biomarker expression to improve the results. Positive and negative results have been reported. Dr Trneny will critically discuss these findings, providing new insights for a possible future molecular driven DLBCL therapy.

